# Cyclin C: A new responser for chemosensitivity in cancer

**DOI:** 10.1002/ctm2.833

**Published:** 2022-04-26

**Authors:** Shuai Fang, Xiaofeng Jin, Chengwei Zhou, Zhaohui Gong

**Affiliations:** ^1^ Department of Thoracic Surgery The Affiliated Hospital of Medical School of Ningbo University Ningbo China; ^2^ Department of Biochemistry and Molecular Biology Ningbo University School of Medicine Ningbo China; ^3^ Zhejiang Province Key Laboratory of Pathophysiology Ningbo University School of Medicine Ningbo China

**Keywords:** cyclin C, chemotherapy, gastric cancer

## Abstract

The resistance to cisplatin‐based chemotherapy is a common cause of poor prognosis in cancer patients. Cisplatin stimulation causes cyclin C translocating to mitochondria, and in turn induces mitochondrial fission. However, little is known about the role of cyclin C in mitochondrial dysfunction in cancer cells challenged with cisplatin. In the present commentary, we bring to the attention of readers the recent report by Jiang et al which revealed the importance of ubiquitylation and translocation of cyclin C in gastric cancer cells in response to cisplatin stimulation for mitochondrial stability. This finding provides new insights into exploring the novel mechanisms of chemoresistance and developing the new chemotherapy synergistic agents in the era of precision oncology.

## 1 BACKGROUND

Gastric cancer is the fifth most common cancer worldwide and the fourth leading cause of cancer‐related death.^1^ Chemotherapy is one of the treatment strategies for patients with cancer, and cisplatin is commonly used as a conventional chemotherapy agent alone or combined with other cytotoxic drugs for advanced gastric cancer.^2^ Despite its success against gastric cancer, the effectiveness of ch is more limited because of acquired or intrinsic resistance. Given that resistance to chemotherapeutic agents is largely attributed to genetic or epigenetic changes,^3^ to determine the biology and mechanism of chemoresistance, and to develop new chemotherapy synergistic agents are vital for the treatment of gastric cancer.

Ubiquitylation, a post‐translational modification that involves a covalent attachment of ubiquitin to a protein substrate, is essential for cellular homeostasis. Aberrant expression, mutations, and deregulated activity of ubiquitylation enzymes have been associated with cancer development and chemoresistance.^4^ And ubiquitylation dysregulation‐mediated protein mitochondrial localization impaired the intrinsic apoptotic pathway and conferred cisplatin resistance to nasopharyngeal carcinoma cells,^5^ but has not been reported in gastric cancer.

Cyclin C (encoded by the *CCNC* gene) is a highly conserved nuclear protein and makes an entry into to cell cycle in the form of cyclin C/Cdk3 complexes as key regulators.^6^ Upon oxidative stress, cyclin C is recruited from nuclei to mitochondria and promotes mitochondrial division and apoptosis of the yeast cell.^7^ A cDNA microarray‐based study found that cyclin C was lowly expressed in tumour tissues compared with normal ones in gastric cancer.^8^ However, the role of cyclin C ubiquitylation in mitochondrial dysfunction in gastric cancer cells challenged with cisplatin remains unclear.

## 2 COMMENTARY

**FIGURE 1:**
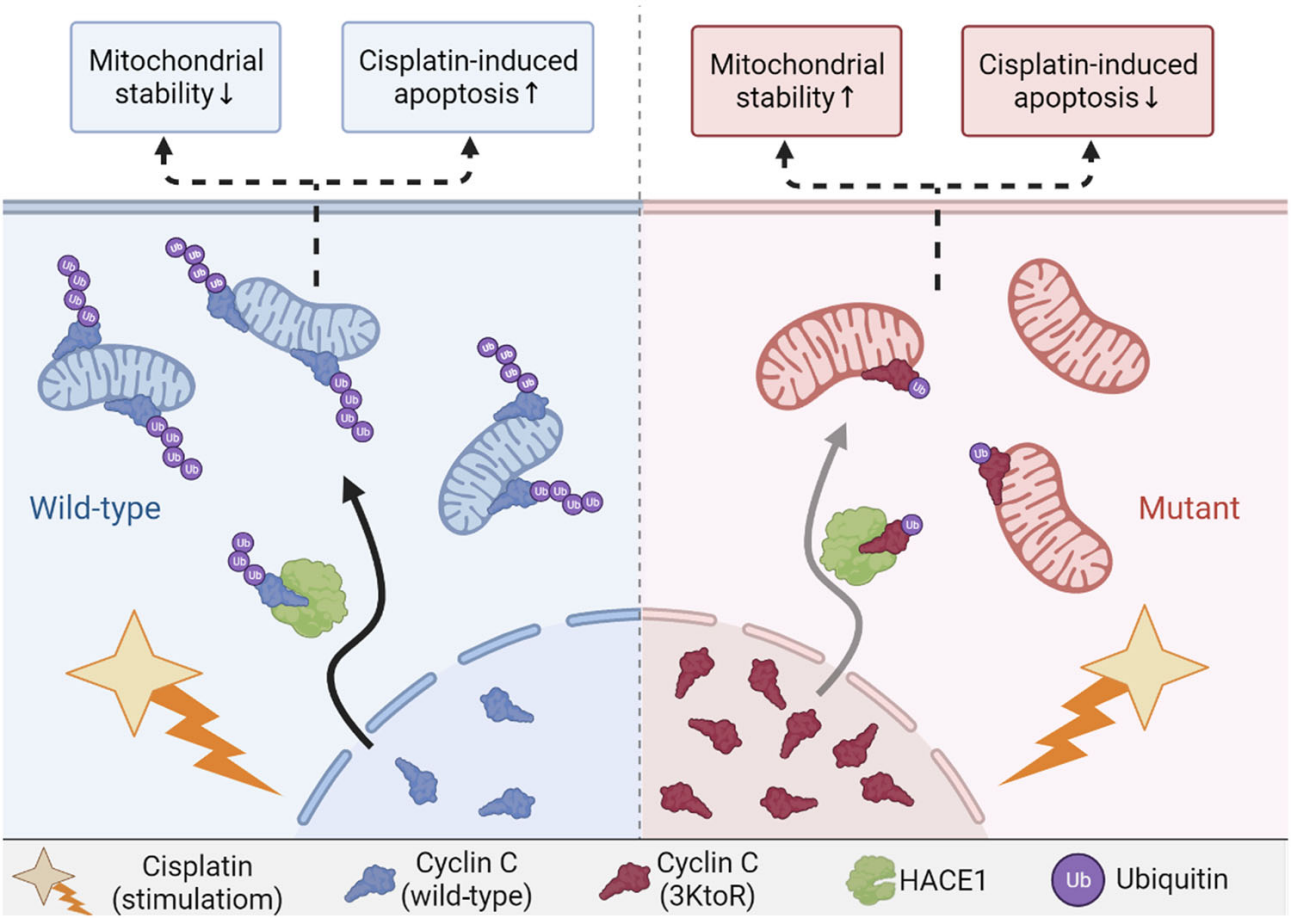
A mechanistic map of dysregulation of cyclin C ubiquitylation to make gastric cancer cells resistant to cisplatin. Ubiquitylation of wild‐type cyclin C by HACE1 followed by translocation from the nucleus to the mitochondria regulates enhanced mitochondrial stability reducing cisplatin‐induced apoptosis, while mutation of three lysine residues to arginine residues (3KtoR) in cyclin C attenuates this effect

Recently, Jiang and colleagues revealed a novel mechanism of HECT domain and ankyrin repeat‐containing E3 ubiquitin‐4 protein ligase 1 (HACE1) ubiquitylation modifying cyclin C to regulate tumour cell resistance to cisplatin in gastric cancer.^9^ The depletion of cyclin C in the gastric cancer HGC27 cell line resulted in high cell viability and mitochondrial respiration levels, as well as minimal apoptosis and mitochondria‐derived reactive oxygen species (ROS) levels in the face of cisplatin treatment. Correspondingly, the overall survival was lower in patients with intestinal and mixed gastric cancer in clinicopathology with lower cyclin C expression. HACE1 is frequently downregulated as an E3 ubiquitin ligase with tumour suppressive effects in multiple cancers including gastric cancer.^10^ In Jiang's work, the cyclin C protein was identified for the first time as a substrate for HACE. Interestingly, the interaction of cyclin C with HACE1 was not detected in HEK‐293FT cells when not exposed to cisplatin. Unlike conventional degradative ubiquitylation, HACE1 mediated non‐degradative ubiquitylation ofcyclin C at K11 upon cisplatin stimulation. In addition, K126, K226 and K236 in cyclin C were major sites of HACE1‐mediated ubiquitylation, and the ubiquitylated cyclin C was transferred from the nucleus to the mitochondria in a time‐ and dose‐dependent manner upon cisplatin stimulation. Furthermore, the mutant cell line (three lysine residues mutated to arginine residues, 3KtoR) could reduce ubiquitylation by HACE1 and translocation to mitochondria, and alleviate cisplatin stress on tumour cells compared with wild‐type cells. Previously, it was reported that cyclin C acted on Mdv1p and Dnm1p to regulate mitochondrial fusion and fission in stressed yeast cells,^7^ and this study confirmed the similar finding in cisplatin‐treated gastric cancer cells, and the mutant 3KtoR cells showed better mitochondrial stability compared with the wild‐type cells (Figure 1).

Although the interaction of HACE1 with cyclin C protein is initially shown to be less related to its N‐terminal ANK1‐6 domain by using a truncation method, the specific binding site is not defined and further studies are needed. Carrying corresponding mutation may be detrimental to the subsequent precise treatment. Of note, there is no obvious hot spot for point mutations of cyclin C ubiquitylation sites in gastric cancer (http://www.cbioportal.org/). However, the differences among cells, malignancies and species make the results of this study far from extrapolation to other tumour types or clinical translational applications.

## 3 CONCLUSION

In summary, although the physiologic role of HACE1‐mediated ubiquitylation of cyclin C is essential for the cisplatin‐induced oxidative stress response and is a novel mechanism for cisplatin‐related drug resistance, the pathologic state of either HACE1 or cyclin C is warranted to be determined in the future. Moreover, given the cellular heterogeneity in the tumour population, the development of a broad spectrum of synergistic chemotherapeutic targets and drugs will be one of the future directions of this work.

## CONFLICT OF INTEREST

The authors declare no conflict of interest.
